# Statistical methodologies for enhancing lipase production from *Aspergillus Niger* and using biologically treated cottonseed waste in animal nutrition

**DOI:** 10.1038/s41598-025-19731-y

**Published:** 2025-10-07

**Authors:** Dina H. El-Ghonemy, Eman M. Abdellah, Mohamed Fadel, Thanaa H. Ali

**Affiliations:** Microbial Chemistry Department, Biotechnology Research Institute, National Research Centre, 33 El Buhouth St., Dokki, 12622 Cairo Egypt

**Keywords:** *Aspergillus Niger* MK377324, Lipase, Statistical designs, Solid-state fermentation, Animal nutrition, Biotechnology, Microbiology

## Abstract

Lipases are industrially valuable biocatalysts; mainly microbial lipases, hence screening and production of lipases from various microbial strains are constantly emerging to meet the needs of the pharmaceutical and food industry sectors. More recently, different cost-effective and efficient techniques to improve lipase synthesis in microbial strains have been investigated. This article attempts to connect the production of lipases from a local isolate *Aspergillus niger* MK377324 under solid-state fermentation with agro-industrial waste as substrate. Plackett-Burman (PB) and central composite statistical designs (CCD) were employed to examine the effect of multiple factors such as carbon and nitrogen sources, pH, and temperature on the production of lipase from *A. niger*. The results showed that cottonseed waste was the optimal substrate for *A. niger* lipase production, while peptone, K_2_HPO_4_, and CuSO_4_ at final concentrations of 5.0, 2.0, 0.5, and 0.015 g/L significantly increased lipase synthesis, yielding 21.42 ± 1.3 U/g ds. Agro-industrial byproducts, which are widely available, can serve an important role in ruminant nutrition. As a result, the residual biomass of the biologically processed cottonseed waste was observed to have high protein content of 29.6% and a considerable percentage of essential amino acids such as lysine (1.28%), therionine (1.04%), and methionine (0.44%), while the crude fibre was decreased from 18.3 to 15.6%, with the potential to significantly improve the nutritional value of animal feed. Overall, using cottonseed waste as a substrate and feed source represents a promising solution to environmental and nutritional challenges. The main advantage of using lipase as a biocatalyst in a variety of organic transformations is that it may also be utilized to produce biodiesel.

## Introduction

Crop waste and agro-industrial byproducts are abundant and play an important role in rumen feeding. By-product use in animal feeding may improve the economics and suitability of industrial, animal, and agricultural production^1^. In many developing countries, there is a gap between available and required animal feed. Some countries purchase cereal grains to increase livestock productivity and meet increased demand for animal products. Plant-derived metabolites containing natural antibiotics and health-promoting compounds can be used in feed additives to address these difficulties^2^. However, the processing of fruits and vegetables generates thousands of tons of agricultural waste and leftovers each year. Although Egypt generates around 22 million tons of plant byproducts each year, only 4.15 million tons of crop residuals are given to animals^3^ due to their difficult digestion, poor feed palatability, low protein, and high fiber content. In Egypt, the gap between animal feed availability and demand is around 9 million tons of dry leftovers, which equates to nearly 4 million tons of total digestible nourishment annually^4^. Many studies are interested in changing unconventional feed sources to solve feed and protein deficits. To address Egypt’s animal feed shortage, efforts must be made to maximize the usage of all available wastes and byproducts^5^. To address this problem, biological, physical, and chemical treatments can be used to increase the nutritional content of agricultural waste^6,7^. Cottonseed hull is a byproduct of cottonseed oil production and makes up around 25–38% of the fuzzy entire cotton seed^8^. According to data on 2020, the cottonseed hull stream might reach 1.5 × 10^9^ kg per year, despite not all cotton seed being used for oil production. Cottonseed hulls are currently and proposed for use as animal feed, fertilizer, mulch, mushroom substrate, packaging material, and in furfural manufacture^9^. Cottonseed meal, a byproduct of cottonseed oil extraction, has tremendous potential as a protein source for pig feed due to its high protein content, high yield, low cost, well-balanced amino acid composition, and ease of accessibility^2^. However, cottonseed meal contains various anti-nutritional substances, including gossypol, that have a negative impact on growth and reproductive performance, resulting in its limited use in pig feed. To optimize the benefits of cottonseed meal and promote its use in pig production, it is critical to have a thorough understanding of its nutritional value and present application^10^.

Lipases are glycerol ester hydrolases that break down carboxyl ester bonds in triglycerides into fatty acids and glycerol. They are found in animals, plants, and microbes and play important roles in lipid deposition, transport, and metabolism^11^. They have been widely used in many industries, including food, pharmaceuticals, detergents, bioenergy, cosmetics, and surfactants, because of their superior performance in esterification, transesterification, and deacetylation reactions, as well as their unique ability to have broad substrate specificity, regiospecificity, and stereospecificity^12^. Lipases produced by microorganisms are primarily extracellular and provide a number of advantages, including high temperature tolerance, quick incubation time, low extraction cost, and the ability to catalyze a wide range of reactions in both aqueous and non-aqueous media^13^. Previous research has shown that media composition affects lipase production from *Burkholderia* sp. HL-10^14^, *Aspergillus* fumigatus^15^, *(A) niger* NRRL-599^16^, and *(B) aryabhattai* SE3-PB^17^. Carbon (galactose, Tween-80, olive oil, or any other inducer oil) and nitrogen sources (tryptone, peptone), pH, incubation time, moisture content, inoculum age, inoculum size, agitation rate, and temperature all had a significant effect on lipase production from different sources^14–17^. As a result, many approaches have been used in experimental statistical designs to optimize these parameters for optimal enzyme synthesis from microorganisms^18^. As a result, the purpose of this study was to identify the critical medium components for lipase synthesis by a local isolate, *A. niger* MK377324, using cottonseed waste as the primary substrate, using plackett-burman (PB) and central composite (CCD) statistical designs. Furthermore, the remaining biomass was assessed for potential use as animal feed.

## Materials and methods

### Microorganism and inoculum preparation

The fungus employed was identified microscopically and genetically as *Aspergillus niger* MK377324^19^ by the Regional Center for Mycology and Biotechnology at El-Azhar University in Egypt. Following seven days of growth, 10 mL of saline H_2_O (0.1% Tween 80) was added to the conidia on slants, and the spores were scraped and agitated to create a homogeneous solution for inoculation.

### Chemicals

The Arabic gum and Triton X-100 were supplied by Acros Organics in New Jersey, USA. The para-nitro-phenyl palmitate (*p*-NPP) and tris-(hydroxymethyl)-amino methane were supplied by Sigma-Aldrich (St. Louis, MO, USA). KH_2_PO_4_, KHPO_4_, KCl, glucose, dextrose, and MgSO_4_.7H_2_O were purchased from Merck in Darmstadt, Germany. The bacteriological peptone was provided by LobaChemie, Mumbai, India.

### Substrate

Cottonseed waste was cleaned under running water, evaporated at 60 °C, milled to a standard size (no. 6 mesh), and stored at room temperature in a plastic bag.

### Detection of aflatoxins

Toxicity testing for chosen strains were carried out using the HPLC method established by the Association of Official Analytical Chemists (AOAC)^20^, as previously explained by El-Ghonemy et al.^19^.

### Optimization of lipase production via multi-factorial designs

#### Plackett–Burmans Tatistical design

In 12 trials, an eleven-variable design (cottonseed waste, olive oil, peptone, sucrose, glucose, NaNO_3_, K_2_HPO_4_, KH_2_PO_4_, MgSO_4,_ NaCl and CuSO_4_) was constructed, with each variable stated at two rates (-1 and + 1). The column denotes the independent variable, whereas the row denotes the test run. The first order linear model indicating the association between enzyme activity and the eleven items was the following:

$${Y_{activity}}={\text{8}}.{\text{321}} - 0.{\text{277}}{X_1}\,+\,{\text{5}}.{\text{16}}{X_2} - 0.{\text{4}}0{\text{2}}{X_6}\,+\,{\text{7}}.{\text{655}}{X_7} - {\text{6}}.{\text{321}}{X_8} - {\text{5}}.{\text{882}}{X_9} - {\text{2}}.{\text{443}}{X_{10}}\,+\,{\text{6224}}{X_{11}}$$

#### Central composite experimental design (CCD)

Four significant variables (Cottonseed waste, peptone, K_2_HPO_4_ and CuSO_4_) were chosen for further analysis with CCD. To accommodate the second-order polynomial model, a 2^4^ factorial design was used, which included 8 trials at the axial point and 6 replication trials in the center. The entire design included 30 runs, with the separated parameters tested at five levels. Triple tests were performed, and the median value of lipase from *Aspergillus niger* MK377324 was used as the variable of reliance (response), indicated as “Y”. The 2nd order polynomial coefficients were calculated and evaluated using the statistical program ‘SPSS’ (version 16) using a 2nd degree polynomial model. Regression analysis resulted in the following polynomial equation:

$$\begin{aligned} {{\text{Y}}_{activity}} & ={\text{ }} - {\text{12}}.{\text{376}}\,+\,{\text{6}}.{\text{367}}{X_1}\,+\,0.{\text{281}}{X_2}\,+\,{\text{7}}.{\text{479}}{X_3} - {\text{55}}0.{\text{446}}{X_4}\,+\,0.{\text{138}}{X_1}^{{\text{2}}} \\ & \;\;\; - {\text{ }}0.{\text{145}}0{X_2}^{{\text{2}}}\,+\,0.0{\text{379}}{X_2}^{{\text{2}}}\,+\,{\text{3}}.{\text{489}}{X_3}^{{\text{2}}}\,+\,{\text{3}}0{\text{6}}{X_4}^{{\text{2}}} - {\text{ }}0.{\text{14}}0{X_1}{X_2} \\ & \;\;\; - {\text{2}}.{\text{714}}{{\text{X}}_{\text{1}}}{{\text{X}}_{\text{3}}} - {\text{ 3}}.{\text{226}}{X_1}{X_4}\,+\,0.{\text{8}}0{\text{9}}{X_2}{X_3}\,+\,{\text{61}}.{\text{976}}{X_2}{X_4} - {\text{ 343}}.{\text{287}}{X_3}{X_4} \\ \end{aligned}$$

### Effect of fungal treatment on cottonseed chemical composition

#### Amino acid analysis

The amino acid content was determined at the Regional Center for Food and Feed (RCFF), Giza, Egypt. Amino acid analysis was performed in accordance with Official Method Analysis (OMA)^21^. The following amino acid analysis was conducted: A 10 milligram protein sample was placed in a conical flask with 5mL of formic acid. The flask was then sealed and placed in an ice bath for 16 h. The flask was filled with sodium bisulfite, and the oxidized mixture was then treated with 25 mL of 6 N HCl. Then it was placed in an oven at 110 °C for 24 h. The volume is then reduced to 5–10 mL by opening with a rotating evaporator under vacuum at 60 °C. The pH was adjusted to 2.2 by adding NaOH solution. The hydrolyzed sample was mixed with the required amount of sodium citrate buffer (pH 2.2). The sample was prepared for analysis after all soluble components had been completely dissolved. The analysis was performed using a high-performance amino acid analyzer (Biochrom 30), with data collected and analyzed using the EZ Chrome software.

#### Fiber analysis

Fiber levels were evaluated at Regional Center for Food and Feed in Giza, Egypt. The filter bag technique was used to analyze crude fiber in feeds (ANKOM)^22^.

#### Protein analysis

The crude protein content was determined using the kjeldahl technique, as recommended by the AOAC in 1990^23^. The research used a conversion factor of 6.25, which is commonly used to compute protein content based on nitrogen concentration assessed using the kjeldahl technique.

#### Carbohydrate analysis

The total carbohydrate content was calculated as glucose using the method given by DuBois et al.^24^. One mL of analyte was pipetted into thick walled tubes containing 20–100 µg carbohydrate. A reagent blank with one mL of water and a set of glucose standards (20–100 µg glucose in one mL) were produced concurrently. Each tube received one mL of a 5% (w/v) phenol solution. After 10 min at room temperature, 5 mL of concentrated sulphuric acid was added, with the acid stream directed onto the liquid’s surface through shaking. The tubes were placed in a 25 °C water bath for 10–20 min, and the produced color was measured at 490 nm.

## Results and discussion

### Detection of aflatoxins

**Fig. 1:**
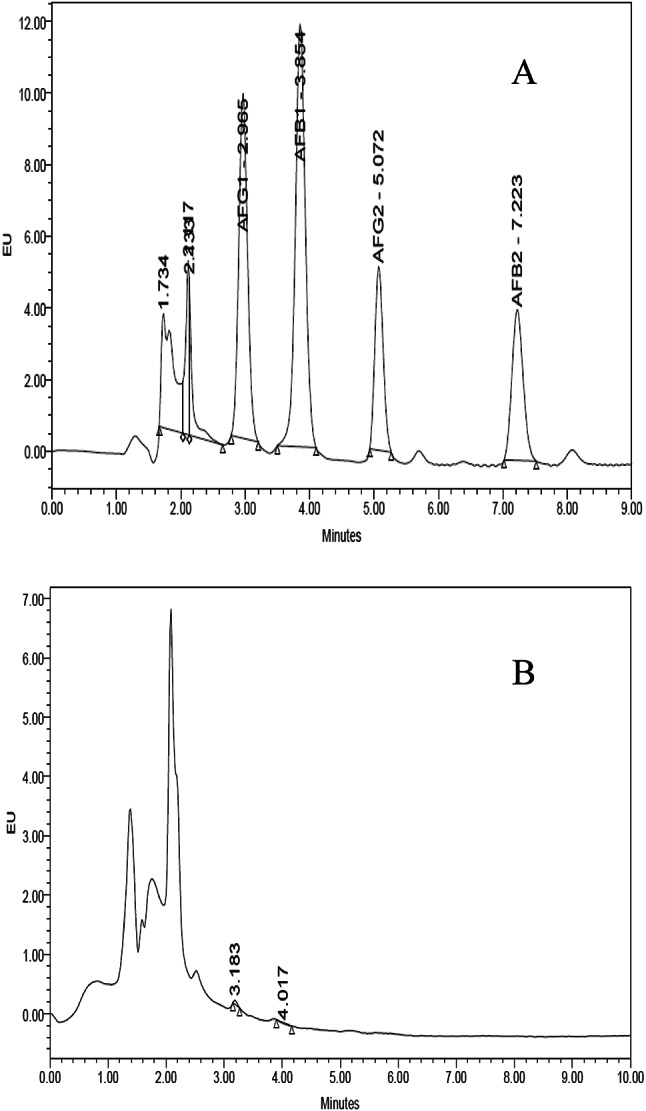
(A) Standard curve of aflatoxins; (B) Aflatoxins test for the highest lipase producer *Aspergillus niger* MK377324 using HPLC technique.

A toxicity test was performed to validate the selection of the fungal isolate with the maximum lipase production, in accordance with AOAC criteria^20^. The results shown in Fig. 1 (A and B) clearly showed that the fungus *Aspergillus niger* MK377324 produced no mycotoxins.

### Multi-factor designs to improve lipase synthesis in A. niger MK377324

The current study shown that *Aspergillus niger* MK377324 produced the highest extracellular lipase of 4.83 ± 0.45 U/g ds when cultivated on cottonseed waste (60% moisture) at pH 7.0 for 6 days at 30 °C. Continuous optimization strategies were used to increase lipase production. The first strategy involves evaluating a large number of dietary characteristics to discover those that had a substantial influence on lipase production. The second strategy tried to maximize the quantities of these components in order to regulate lipase biosynthesis.

### Characterization of fermentation parameters that affect lipase biosynthesis

**Fig. 2:**
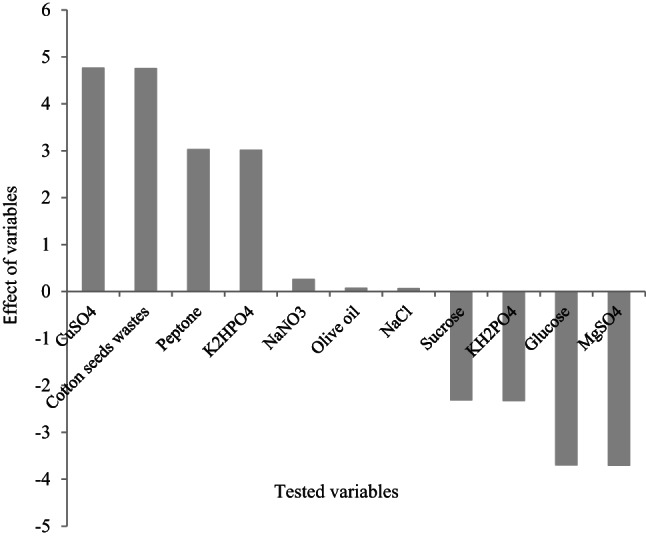
Pareto chart showing the effect of tested variables on lipase production by *Aspergillus niger* MK377324 using cottonseed waste as substrate under SSF.

A PB design was used to show the relevance of various nutritional components on lipase synthesis by *Aspergillus niger* MK377324 grown on cottonseed waste utilizing the solid state fermentation (SSF) technology. For evaluation, 11 medium components were chosen, and the average lipase values were obtained for each trial (Table 1). The impact of each parameter on lipase production was determined by comparing average data at high (+ 1) and low (− 1) levels. The data in Table 1 showed that the maximum and minimum lipase values were 16.39 *±* 0.83 U/g ds and 2.05 *±* 0.03, respectively, in runs 4 and 7, demonstrating the significant effect of medium constituents on lipase synthesis by *A. niger* when cottonseed waste was used as the substrate. The effect of each parameter on lipase synthesis was investigated further and represented in Fig. 2, which clearly shows that peptone, K_2_HPO_4_, CuSO_4_, and cottonseed waste had a beneficial effect on lipase biosynthesis, but MgSO_4_, KH_2_PO_4_, sucrose, and glucose had a negative impact.

**Table 1:** Randomized PB experimental design of cottonseed waste showed coded and un-coded variables levels for evaluating its influencing on lipase production from *Aspergillus Niger* MK377324.

Trial No.	Substrate	Olive oil	Glucose	Sucrose	Peptone	NaNO_3_	K_2_HPO_4_	KH_2_PO_4_	MgSO_4_	NaCl	CuSO_4_	Lipase activity
	X_1_	X_2_	X_3_	X_4_	X_5_	X_6_	X_7_	X_8_	X_9_	X_10_	X_11_	U/gds
1	-1(2)	1(30)	-1(1)	1(3)	-1(2)	1(4)	-1(0.5)	1(1)	-1(0.1)	1(0.5)	-1(-)	8.08 *±* 0.06
2	1(5)	1(30)	-1(1)	-1(1)	-1(2)	1(4)	-1(0.5)	-1(0.5)	-1(0.1)	1(0.5)	1(0.001)	8.20 *±* 0.06
3	1(5)	1(30)	1(3)	-1(1)	-1(2)	-1(2)	-1(0.5)	-1(0.5)	1(0.5)	1(0.5)	1(0.001)	11.37 *±* 0.11
4	1(5)	-1(10)	1(3)	-1(1)	1(4)	1(4)	1(1)	-1(0.5)	1(0.5)	-1(0.1)	1(0.001)	16.39 *±* 0.83
5	-1(2)	1(30)	1(3)	1(3)	1(4)	1(4)	1(1)	1(1)	1(0.5)	1(0.5)	-1(-)	5.61 *±* 0.04
6	1(5)	1(30)	-1(1)	1(3)	1(4)	-1(2)	1(1)	1(1)	-1(0.1)	1(0.5)	1(0.001)	15.34 *±* 0.41
7	1(5)	-1(10)	1(3)	1(3)	-1(2)	1(4)	-1(0.5)	1(1)	1(0.5)	-1(0.1)	1(0.001)	2.05 *±* 0.03
8	-1(2)	1(30)	1(3)	-1(1)	1(4)	-1(2)	1(1)	-1(0.5)	1(0.5)	1(0.5)	-1(-)	1.26 *±* 0.02
9	1(5)	-1(10)	-1(1)	1(3)	1(4)	-1(2)	1(1)	1(1)	-1(0.1)	-1(0.1)	1(0.001)	9.40 *±* 0.08
10	-1(2)	-1(10)	1(3)	1(3)	-1(2)	-1(2)	-1(0.5)	1(1)	1(0.5)	-1(0.1)	-1(-)	2.83 *±* 0.04
11	-1(2)	-1(10)	-1(1)	-1(1)	1(4)	1(4)	1(1)	-1(0.5)	-1(0.1)	-1(0.1)	-1(-)	10.05 *±* 0.09
12	-1(2)	-1(10)	-1(1)	-1(1)	-1(2)	-1(2)	-1(0.5)	-1(0.5)	-1(0.1)	-1(0.1)	-1(-)	8.78 *±* 0.07

The first linear order model, which illustrates the link between the eleven parameters and enzyme synthesis, is shown below:

Table 2 shows that CuSO_4_ had the highest effect, followed by cottonseed waste, peptone, and K_2_HPO_4_, as demonstrated by *t*-tests (0.58, 0.58, 0.37, and 0.37, respectively) and highly significant *p*-values (0.02, 0.02, 0.03, and 0.03, respectively). Based on our findings, it is feasible to use CuSO_4_, cottonseed waste, peptone, and K_2_HPO_4_ as essential parameters for further optimization using Central composite statistical design (CCD). Our findings exceed those of Salihu et al.^25^, who evaluated eleven medium components and identified Na_2_HPO_4_, (NH_4_)_2_SO_4_, MgSO_4_, Tween-80, sugar, and olive oil as positive contributions to total lipase synthesis, with a maximum production of 3.35 U/g. Furthermore, Rajendran and Thangavelu^26^ found that peptone, MgSO_4_.7H_2_O, KH_2_PO_4_, CaCl_2_.H_2_O and olive oil were beneficial variables in *Rhizopus arrhizus* producing the maximum lipase level of 3.98 U/mL. It is widely understood that glucose catabolic repression can impair lipase enzyme function in fungi such as *Fusarium oxysporum*, *A. niger*, *Rhizopus delemar*, and *Rhizomucor miehei*^27^. Mehta et al.^15^ investigated the effect of six variables on lipase production by *A. fumigatus* and observed that galactose, peptone, pH, and incubation time were important variables to consider in PB design. They concluded that 1.5% galactose, 1.8% peptone, a pH of 10.0, and a 72-h incubation period at 45 °C were appropriate under response surface curves, with a coefficient of determination (R^2^) of 0.9318 indicating that the model was significant.

**Table 2:** Statistical analysis of PB design showing coefficient values, t- and P- values for each variable on *Aspergillus Niger* MK377324 lipase activity on cottonseed waste.

Variables	Coefficient	t-statistics	*P*-value	Confidence level (%)
Intercept	8.321			
Cottonseed waste	-0.277	0.584748	0.02858	97.142
Olive oil	0	0.008544	0.4967	50.33
Glucose	0	-0.45489	0.3295	67.05
Sucrose	0	-0.28438	0.391	60.9
Peptone	0	0.372396	0.03587	96.413
NaNO_3_	-0.402	0.031719	0.04877	95.123
K_2_HPO_4_	7.655	0.370692	0.037069	96.29308
KH_2_PO_4_	-6.321	-0.28613	0.3903	60.97
MgSO_4_	-5.882	-0.45581	0.3291	67.09
NaCl	-2.443	0.007611	0.497	50.3
CuSO_4_	6224	0.586308	0.02853	97.147

### Central composite statistical design

CCD was used to determine the optimal concentrations of the most important factors for lipase synthesis. Table 3 shows the coded and un-coded values of the four selected independent factors, the CCD program, and the observed and expected lipase yields. A multiple regression analysis of the experimental results produced the following second order polynomial equation:

**Table 3:** CCD using cottonseed waste consisting of 30 experiments for four experimental factors in coded and actual values for the production of lipase by *Aspergillus Niger* MK377324.

Trial No.	Substrate X1		Peptone X2		K_2_HPO_4_ X3		CuSO_4_ X4		Lipase activity (U/gds)	
	Coded	Actual (g/L)	Coded	Actual (g/L)	Coded	Actual (g/L)	Coded	Actual (g/L)	Experimental	Predicted
1	-1	3	-1	2	-1	0.5	-1	0.005	4.60 *±* 0.05	6.214
2	-1	3	1	4	-1	0.5	-1	0.005	5.85 *±* 0.08	5.625
3	0	4	0	3	0	0.75	+ ∞	0.02	13.80 *±* 0.30	14.432
4	0	4	0	3	0	0.75	0	0.01	9.16 *±* 0.15	11.239
5	0	4	0	3	0	0.75	- ∞	0.001	14.45 *±* 0.36	13.804
6	-1	3	1	4	1	1	1	0.015	9.03 *±* 0.14	8.476
7	0	4	0	3	0	0.75	0	0.01	9.44 *±* 0.16	11.239
8	-1	3	1	4	-1	0.5	1	0.015	6.97 *±* 0.10	7.148
9	0	4	0	3	0	0.75	0	0.01	12.72 *±* 0.28	11.239
10	-1	3	1	4	1	1	-1	0.005	5.75 *±* 0.07	8.670
11	1	5	-1	2	-1	0.5	-1	0.005	17.25 *±* 0.52	17.850
12	0	4	0	3	0	0.75	0	0.01	14.67 *±* 0.38	11.239
13	1	5	1	4	1	1	-1	0.005	17.66 *±* 0.55	17.032
14	1	5	1	4	1	1	1	0.015	18.89 *±* 0.99	16.774
15	-1	3	-1	2	-1	0.5	1	0.015	5.75 *±* 0.07	6.498
16	0	4	0	3	- ∞	0.25	0	0.01	11.08 *±* 0.25	11.686
17	1	5	1	4	-1	0.5	-1	0.005	18.89 *±* 0.99	16.701
18	1	5	-1	2	-1	0.5	1	0.015	21.42 *±* 1.3	18.069
19	+ ∞	6	0	3	0	0.75	0	0.01	18.48 *±* 0.96	21.758
20	- ∞	2	0	3	0	0.75	0	0.01	4.71 *±* 0.05	1.825
21	-1	3	-1	2	1	1	-1	0.005	9.44 *±* 0.16	8.450
22	0	4	- ∞	1	0	0.75	0	0.01	10.67 *±* 0.20	10.504
23	0	4	0	3	0	0.75	0	0.01	13.55 *±* 0.32	11.239
24	1	5	1	4	-1	0.5	1	0.015	17.05 *±* 0.50	18.159
25	0	4	0	3	0	0.75	0	0.01	7.60 *±* 0.11	11.239
26	-1	3	-1	2	1	1	1	0.015	5.33 *±* 0.07	7.0175
27	0	4	0	3	+ ∞	1.5	-1	0.005	16.01 *±* 0.45	15.916
28	1	5	-1	2	1	1	0	0.01	17.05 *±* 0.50	15.828
29	0	4	+ ∞	5	0	0.75	0	0.01	10.26 *±* 0.19	10.814
30	1	5	-1	2	1	1	1	0.015	14.99 *±* 0.40	15.874

$$\begin{aligned} {{\text{Y}}_{activity}} & ={\text{ }} - {\text{12}}.{\text{376}}\,+\,{\text{6}}.{\text{367}}{X_1}\,+\,0.{\text{281}}{X_2}\,+\,{\text{7}}.{\text{479}}{X_3} - {\text{55}}0.{\text{446}}{X_4}\,+\,0.{\text{138}}{X_1}^{{\text{2}}} \\ & \;\;\; - {\text{ }}0.{\text{145}}0{X_2}^{{\text{2}}}\,+\,0.0{\text{379}}{X_2}^{{\text{2}}}\,+\,{\text{3}}.{\text{489}}{X_3}^{{\text{2}}}\,+\,{\text{3}}0{\text{6}}{X_4}^{{\text{2}}} - {\text{ }}0.{\text{14}}0{X_1}{X_2} \\ & \;\;\; - {\text{2}}.{\text{714}}{{\text{X}}_{\text{1}}}{{\text{X}}_{\text{3}}} - {\text{ 3}}.{\text{226}}{X_1}{X_4}\,+\,0.{\text{8}}0{\text{9}}{X_2}{X_3}\,+\,{\text{61}}.{\text{976}}{X_2}{X_4} \\ & \;\;\; - {\text{ 343}}.{\text{287}}{X_3}{X_4} \\ \end{aligned}$$

Where Y _activity_: is the response (lipase production); X1, X2, X3, and X4: are the coded values of the variables examined (cottonseed waste, peptone, K_2_HPO_4_ and CuSO_4_, respectively).

**Fig. 3:**
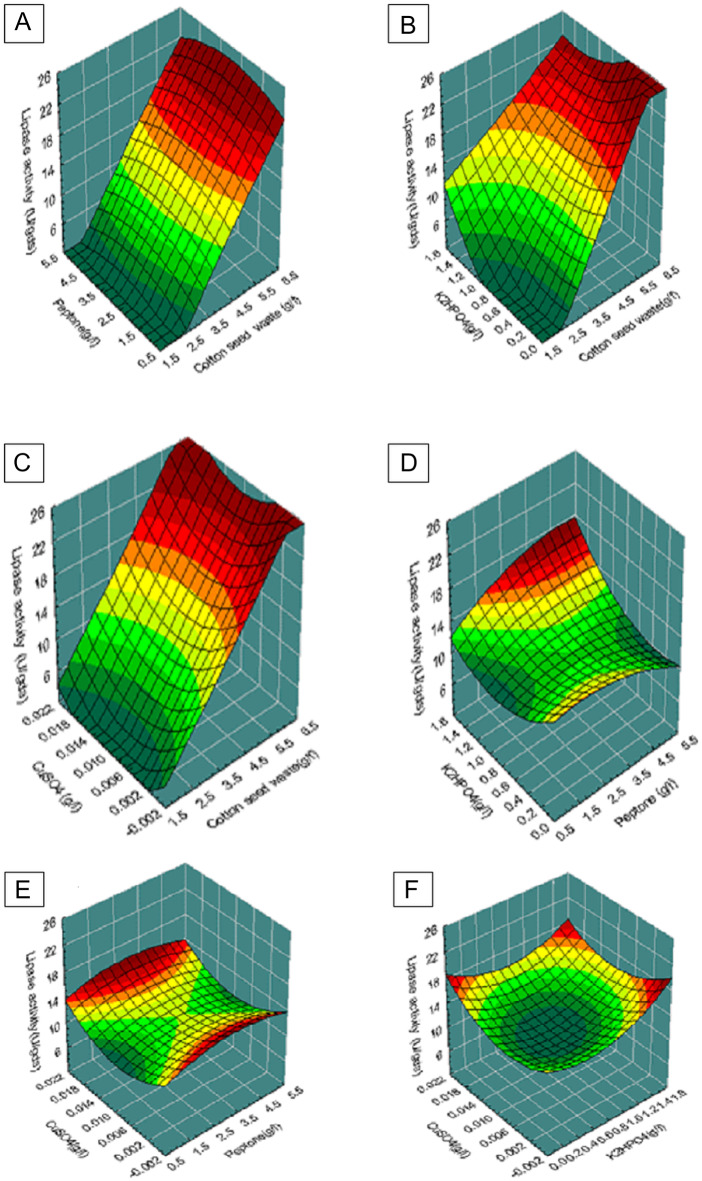
(A-F) Response surface plot of lipase production by *Aspergillus niger* MK377324 showing the interaction between different concentrations of: (A) cottonseed waste and peptone at X_3_ = 0 and X_4_ = 0; (B) cottonseed waste and K_2_HPO_4_ at X_2_ = 0 and X_4_ = 0; (C) cottonseed waste and CuSO_4_ at X_2_ = 0 and X_3_ = 0; (D) peptone and K_2_HPO_4_ at X_1_ = 0 and X_4_ = 0; (E) peptone and CuSO_4_ at X_1_ = 0 and X_3_ = 0; (F) K_2_HPO_4_ and CuSO_4_ at X_1_ = 0 and X_2_ = 0.

The regression analysis of the CCD data enabled the creation of 3D response graphs revealing the interaction of the most significant variables in lipase production (Fig. 3 (A-F)). Similar to the findings of Mehta et al.^15^, the response surface graphs in Fig. 3 in our investigation showed a hill-shaped pattern, showing the presence of interactions between variables that led to maximal enzyme activity. Chien-Hung et al.^28^ discovered that the coefficient (*R*^*2*^) was 0.81, indicating that the model was significant with the lipase production values produced by *Burkholderia* sp. The ANOVA analysis findings (Table 4) revealed a substantial *F*-value (6.634), indicating that the statistical model is significant. Model terms with *P > F* (0.00001) values of less than 0.05 are considered important. The model’s quality was evaluated by computing the coefficient (*R*^2^) and the coefficient of correlation (*R*). The closer the *R*^*2*^ is to one, the more effective the model and greater the expected response. In the current investigation, the coefficient (*R*^2^) for lipase production was estimated to be 0. 861 (*R*^*2*^ > 0.75 denotes model adequacy), meaning that the statistical model can explain 86.1% of the response variation.

**Table 4:** ANOVA and model coefficients estimated by multiples linear regression (significance of regression coefficients).

Variables	Regression coefficients	Standard error	t- test	*P*-value
Intercept	-12.376	18.631	-0.664	0.517
X_1_	6.367	5.066	1.257	0.228
X_2_	0.281	4.583	0.061	0.952
X_3_	7.479	17.802	0.420	0.680
X_4_	-550.446	878.937	-0.626	0.541
X_11_	0.138	0.501	0.275	0.787
X_22_	-0.145	0.501	-0.288	0.777
X_33_	3.489	5.703	0.612	0.550
X_44_	31806.595	21782.947	1.460	0.165
X_12_	-0.140	0.657	-0.213	0.834
X_13_	-2.714	2.630	-1.032	0.318
X_14_	-3.226	137.362	-0.023	0.982
X_ute_	0.809	2.630	0.308	0.763
X_sti_	61.976	137.362	0.451	0.658
X_ste_	-343.287	516.816	-0.664	0.517

ANOVAs					
	Df	SS	SM	*F* test	Significance *F* (P)
Regression	14	627.641	44.832	6.634	0.00001
Residual	15	101.372	6.758		
Total	29	729.013			

Df: Degree of freedom; SS: Sum of squares; MS: Mean sum of squares; F: Fishers’s function; Significance F: corresponding level of significance. R^2^ = 0.861 Adjusted R^2^ = 0.

The biosynthesis of lipase increased by 4.4-fold when *Aspergillus niger* MK377324 was grown on cottonseed waste medium (pH 7.0) enriched with cottonseed waste, peptone, K_2_HPO_4_ and CuSO_4_ at concentrations of 5.0, 2.0, 0.5, and 0.015 g/L for 6 days at 30 °C using SSF. As a result, our data clearly proved the ability of *A. niger* to produce extracellular lipase under SSF conditions, as well as the proposed model’s effectiveness. In this concern Kaushik et al.^29^ reported that the maximal lipase activity achieved was 12.7 U/ml utilizing the CCD experiment for each individual run, as predicted^29^. Additionally, Al-Khattaf et al.^30^ found that the central composite design increased lipase synthesis by *A. niger* LP4 by 2.1-fold compared to the basal medium. The F-value of the constructed model was 12.98, with a p-value of 0.0002. Kaur and Gupta^31^ investigated extracellular lipase synthesis from a thermotolerant *Bacillus subtilis* TTP-06. ANOVA findings with a p-value of < 0.05 revealed an *R*^*2*^ value of 0.7846.

### Evaluation of the residual biomass as nourishment for animals

#### Amino acid analysis

**Fig. 4:**
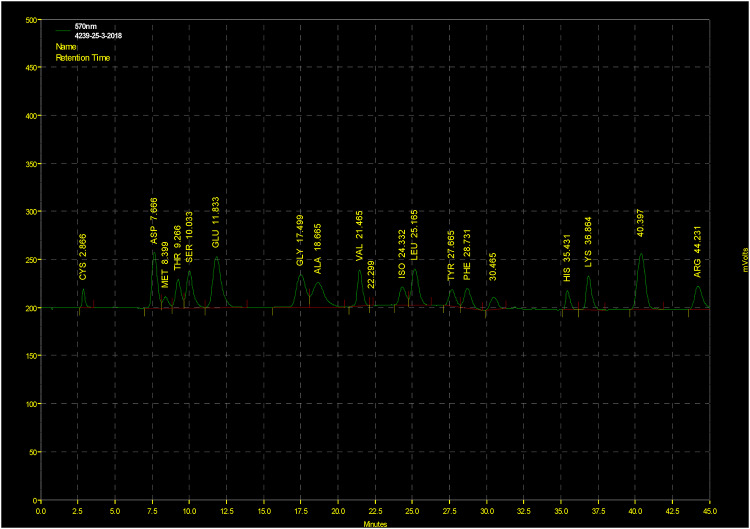
Amino acid analysis of cottonseed waste treated with *Aspergillus niger* MK377324.

Table 5; Fig. 4 illustrate the amino acid content of cottonseed waste following *Aspergillus niger* MK377324 growth. According to this study, using cottonseed waste as a substrate resulted in a considerable increase in methionine (METH), therionine (THR), and lysine (LYS). The percentages of these amino acids were METH (0.44%), THR (1.04%), and LYS (1.28%).

**Table 5:** Amino acid analysis of treated cottonseed waste.

Amino acid	Cottonseed waste		
	Retention time (RT)	Amino acid (%)	AA % in crude protein
Cysteine (cys)	2.866	0.34	1.14
Aspartic (ASP)	7.666	2.10	7.09
Methionine (met)	8.399	0.44	1.48
Therionine (THR)	9.266	1.04	3.51
Serine (SER)	10.033	1.12	3.78
Glutamic (GLU)	11.833	3.19	10.7
Glycine (GLY)	17.499	1.14	3.85
Alanine (ALA)	18.665	1.42	4.79
Valine (VAL)	21.465	1.20	4.05
Isoleucine (ILE)	24.332	0.85	2.87
Leucine (LEU)	25.165	1.50	5.06
Tyrosine (TYR)	27.665	0.93	3.14
Phenylalanine (PHE)	28.731	1.10	3.71
Histidine (HIS)	35.431	0.63	2.12
Lysine (LYS)	36.864	1.28	4.32
Proline (PRO)	40.397	1.02	3.44
Argnine (ARG)	44.231	1.62	5.47

#### Effect of fungal treatment on the chemical composition of cottonseed

Plant-derived metabolites containing natural antibiotics and health-promoting compounds can be used in feed additives. Cottonseed, a byproduct of the cotton plant (*Gossypium* spp.), is a promising source of fat and protein^2^. According to a pre-treatment chemical research, cottonseed waste can be used for animal and poultry nutrition. It includes 26.1% protein, 18.3% fiber, 51.3% carbohydrate, and only 4.3% ash. Low lignin content is particularly favorable to microbial growth and replication. The growth of *Aspergillus niger* MK377324 had a significant impact on the chemical composition of cottonseed waste based on dry weight, with treated waste having a higher percentage of protein and ash than untreated waste (Raw). The crude protein (CP) content of biologically processed cottonseed waste increased from 26.1 to 29.6% (Table 6). Animal feed is frequently supplemented with vitamins, trace elements, and minerals to improve its nutritional value. The supplementation of essential amino acids such as METH, LYS, and THR is critical for enhancing animal growth and productivity, resulting in more meat and milk production per kilogram of feed. This method helps to preserve precious plant resources. Global production of lysine for the feed operation exceeds one billion tons annually. Lysine synthesis through chemical means is not economically viable. Consequently, biotechnologists have engineered microorganisms to synthesis amino acids on an industrial scale. This is done in large steel containers where the bacteria can multiply under ideal conditions^32^.

**Table 6:** Chemical composition of cottonseed waste before and after fungal treatment.

Analysis	Content (%) Before growth	Content (%) After growth
Crude protein	26.1	29.60
Crude fiber	18.30	15.64
Ash	4.30	6.86
Carbohydrate	51.30	47.9

Improving feed efficiency in pigs by the dietary use of amino acids is becoming increasingly important. This approach not only provides an appropriate supply of plasma amino acids for muscle growth, but it also lowers nitrogen release into the environment via urine and feces. Lysine, the first restricted amino acid in standard pig nutrition, acts as an intermediary in the production of body proteins, peptides, and non-peptide substances. Lysine is broken down in abundance to provide energy. Lysine is essential for controlling amino acid metabolism and can affect the metabolism of other nutrients^30^. The CP content is consistent with that of Iyayi and Aderolu^33^, who found an increase in CP levels in agro-wastes treated with *Trichoderma viride*. Furthermore, Dhanda et al.^34^ found that biological treatment enhanced the CP content of spent straw from 3.42 to 6.19%. Dietary fiber is made up of non-starch polysaccharides such as arabinoxylans, cellulose, and many other plants, including resistant dextrins, inulin, waxes, chitins, pectins, glucan, and oligosaccharides, which are considered to be anti-nutritional for many animals. Cellulases successfully hydrolyze cellulose in feed ingredients, converting it into a readily absorbable substance that improves animal health and performance^35^. According to Karau and Grayson^36^, proteins are made up of twenty amino acids. Some of these, known as essential amino acids, must be included in animal feed since animals cannot produce them. The essential amino acids may differ depending on the species. Feeds must provide essential amino acids in sufficient quantities and meet the livestock’s needs in order to maintain maximum health and growth performance. Cottonseed waste has been identified as a promising source of proteinaceous nutrients as a result of the growing emphasis on cost reduction in industrial operations and the need to add value to agro-industrial residues. It can serve as a support matrix for a variety of biotechnological operations. As a result, cottonseed waste can be used as an alternative feedstock in ruminant diets to reduce pollution and feeding expenses. As a consequence, *A. niger* MK377324 treatment of cottonseed waste can improve its nutritional value while having no effect on animal performance since the nutrient content of the treated cottonseed waste was improved by increasing total protein, decreasing crude fibers, and increasing total energy.

## Conclusion

Microbial lipases are one of the most abundant enzymes. They have numerous uses and benefits in the food and agriculture industries. Microbial lipases can be produced by utilizing both solid-state and submerged fermentation techniques using a variety of carbon and nitrogen sources. However, the majority of the literature reports agreed that solid state fermentation is the better method for producing microbial lipase. In this study, the nutritional parameters influencing lipase production by *Aspergillus niger* MK377324 have been investigated and found to be important in increasing lipase yield using cottonseed waste as substrate and peptone, K_2_HPO_4_, and CuSO_4_ at final concentrations of 5.0, 2.0, 0.5, and 0.015 g/L, respectively, under SSF. Furthermore, the treated residual biomass of cottonseed waste had high protein content of 29.6% and a considerable percentage of essential amino acids, which has the potential to significantly improve the nutritional value of animal feed, while the crude fibre and carbohydrate were decreased from 18.3% and 51.3–15.6% and 47.9%, respectively. This has the potential to boost animal milk and meat production, solving both environmental concerns and animal feed scarcity. Overall, using cottonseed waste as a substrate and feed source represents a promising solution to environmental and nutritional challenges. The main advantage of using lipase as a biocatalyst in a variety of organic transformations is that it may also be utilized to produce biodiesel.

## Data Availability

All data generated or analysed during this study are included in this published article.
